# Procedural and 1-year outcomes following large vessel coronary artery perforation treated by covered stents implantation: Multicentre CRACK registry

**DOI:** 10.1371/journal.pone.0249698

**Published:** 2021-05-12

**Authors:** Wojciech Wańha, Rafał Januszek, Michalina Kołodziejczak, Łukasz Kuźma, Mateusz Tajstra, Tomasz Figatowski, Malwina Smolarek-Nicpoń, Monika Gruz-Kwapisz, Brunon Tomasiewicz, Jerzy Bartuś, Andrzej Łoś, Dariusz Jagielak, Tomasz Roleder, Adrian Włodarczak, Jan Kulczycki, Mariusz Kowalewski, Damian Hudziak, Paweł Stachowiak, Jarosław Gorący, Katarzyna Sierakowska, Krzysztof Reczuch, Miłosz Jaguszewski, Sławomir Dobrzycki, Grzegorz Smolka, Stanisław Bartuś, Andrzej Ochała, Mariusz Gąsior, Wojciech Wojakowski

**Affiliations:** 1 Department of Cardiology and Structural Heart Diseases, Medical University of Silesia, Katowice, Poland; 2 Second Department of Cardiology, Jagiellonian University Medical College, Krakow, Poland; 3 Department of Anaesthesiology and Intensive Care, Ludwik Rydygier Collegium Medicum, Nicolaus Copernicus University, Antoni Jurasz University Hospital No. 1, Bydgoszcz, Poland; 4 Department of Invasive Cardiology, Medical University of Bialystok, Bialystok, Poland; 5 Third Department of Cardiology, Medical University of Silesia, Zabrze, Poland; 6 First Department of Cardiology, Medical University of Gdansk, Gdansk, Poland; 7 Department of Heart Disease, Wroclaw Medical University, Poland; 8 Department of Cardiac and Vascular Surgery, Medical University of Gdansk, Gdansk, Poland; 9 Regional Specialist Hospital, Research and Development Center, Wroclaw, Poland; 10 Department of Cardiology, Miedziowe Centrum Zdrowia, Lubin, Poland; 11 Department of Cardiac Surgery, Central Clinical Hospital of the Ministry of Interior, Centre of Postgraduate Medical Education, Warsaw, Poland; 12 Thoracic Research Centre, Collegium Medicum, Nicolaus Copernicus University, Innovative Medical Forum, Bydgoszcz, Poland; 13 Department of Cardiac Surgery, Medical University of Silesia, Katowice, Poland; 14 Department of Cardiology, Pomeranian Medical University, Szczecin, Poland; Baylor Scott and White, Texas A&M College of Medicine, UNITED STATES

## Abstract

**Background:**

Data regarding the clinical outcomes of covered stents (CSs) used to seal coronary artery perforations (CAPs) in the all-comer population are scarce. The aim of the CRACK Registry was to evaluate the procedural, 30-days and 1-year outcomes after CAP treated by CS implantation.

**Methods:**

This multicenter all-comer registry included data of consecutive patients with CAP treated by CS implantation. The primary endpoint was the composite of major adverse cardiac events (MACEs), defined as cardiac death, target lesion revascularization (TLR), and myocardial infarction (MI).

**Results:**

The registry included 119 patients (mean age: 68.9 ± 9.7 years, 55.5% men). Acute coronary syndrome, including: unstable angina 21 (17.6%), NSTEMI 26 (21.8%), and STEMI 26 (21.8%), was the presenting diagnosis in 61.3%, and chronic coronary syndromes in 38.7% of patients. The most common lesion type, according to ACC/AHA classification, was type C lesion in 47 (39.5%) of cases. A total of 52 patients (43.7%) had type 3 Ellis classification, 28 patients (23.5%) had type 2 followed by 39 patients (32.8%) with type 1 perforation. Complex PCI was performed in 73 (61.3%) of patients. Periprocedural death occurred in eight patients (6.7%), of which two patients had emergency cardiac surgery. Those patients were excluded from the one-year analysis. Successful sealing of the perforation was achieved in 99 (83.2%) patients. During the follow-up, 26 (26.2%) patients experienced MACE [7 (7.1%) cardiac deaths, 13 (13.1%) TLR, 11 (11.0%) MIs]. Stent thrombosis (ST) occurred in 6 (6.1%) patients [4(4.0%) acute ST, 1(1.0%) subacute ST and 1(1.0%) late ST].

**Conclusions:**

The use of covered stents is an effective treatment of CAP. The procedural and 1-year outcomes of CAP treated by CS implantation showed that such patients should remain under follow-up due to relatively high risk of MACE.

## Introduction

Coronary artery perforations (CAPs) can occur during percutaneous coronary intervention (PCI) in 0.17%–0.43% of patients presenting with coronary artery disease [1, 2]; the incidence can rise up to 4.1–4.8% in complex procedures on calcified, chronically occluded vessels [3–5]. Almost 39% of CAPs occur at post dilatation stage, during the vessel preparation and stent optimization [6]; direct stenting is correlated with even a higher risk of CAP [5]. For non-severe perforation, conventional treatments (long-term balloon inflation or additional stent implantation) can be used. On contrary, they can also result in proximal massive artery rupture and subsequent life-threatening complications, including acute or late cardiac tamponade (up to a third of patients), which can lead to hemodynamic instability and sudden death. In such cases, the treatment must be prompt and effective. Usually, the covered stent (CS) implantation is the first-line treatment followed by cardiac surgery, if the bleeding persists [7]. The use of CS is an effective strategy for treating CAPs with favorable event-free survival [8]. The CS consists of a metallic stent platform covered with a synthetic or biological membrane that seals the blood extravasation. On the other hand, the material delays endothelialization and is potentially thrombogenic [9, 10]. The Swedish Coronary Angiography and Angioplasty Registry (SCAAR) showed that patients receiving CS have a significantly higher risk of adverse events as compared with drug-eluting stents [11]. However, data about clinical outcomes of CAP treated by CS implantation are scarce. Although the devices have been used for many years, only a small number of studies described periprocedural and long-term outcomes. In the current report we present procedural, in-hospital, and one-year results of patients treated with the CS implantation for iatrogenic, peri-PCI CAP.

## Methods

The covered stent CAP (CRACK) Registry is a large multicenter, retrospective, observational study conducted in high-volume PCI centers. The dataset included consecutive patients with iatrogenic, peri-PCI CAP treated with CS implantation between January 2009 and October 2019. Patients who died during periprocedural period were excluded from the one-year analysis. The study’s angiographic data included in the study were collected, anonymized, and recorded in the central cardiovascular information registry. Outcome data were obtained from the central database of the National Health Fund Service of the Ministry of Health, and no patient was lost to follow-up. In case of re-PCI or coronary artery bypass grafting during the follow-up period, we additionally checked target vessel revascularization and target lesion revascularization (TLR). The patients’ data were fully anonymized in each center, combined into the database, and statistically analyzed together as a single cohort. The study was approved by the Ethics Committee of the Medical University of Silesia. The patient’s data was protected according to the requirements of Polish law, GDPR, and hospital Standard Operating Procedures. The study was conducted in accordance with the Declaration of Helsinki and was registered at ClinicalTrials.gov (NCT04630314).

### Procedure

CS implantation and the type and duration of antithrombotic treatment was at the operator’s discretion, in accordance with the recommendations of the clinical practice guidelines [12]. All stents were implanted into a vessel of minimum 2 mm diameter and were delivered to the perforated segment.

### Angiography analysis

The morphology of the stented lesion was defined according to the classification proposed by the ACC/AHA (American College of Cardiology/American Heart Association) [13]. Lesion length, percentage diameter stenosis, and the coronary flow (thrombolysis in myocardial infarction—TIMI classification) were assessed in all patients. The three-stage Ellis classification was used to determine the degree of perforation based on the angiographic manifestation [14].

### Patient follow-up and study endpoints

The primary endpoint was the composite of major adverse cardiac events (MACE) defined as cardiac death, TLR, and myocardial infarction (MI) assessed after 30 days and one year from the index procedure. Secondary endpoints were stent thrombosis (ST) and the individual events of the primary composite endpoint. TLR was defined as any revascularization procedure within the treated lesion. ST was defined as acute (0-24h post stent implantation), subacute (from 24h to 30 days post stent implantation), or late (from 30 days to 1 year after stent implantation) [15]. Successful perforation sealing was defined as no contrast leak, and no cardiac surgery intervention needed after CS implantation.

### Statistical analysis

Baseline clinical characteristics data are shown as means and standard deviation or numbers and percentages. Kaplan-Meier curves were used to present the unadjusted time-to-event data for MACE. Categorical data were analyzed with the Chi-square or Fisher’s exact test. The statistical analysis was performed using Medcalc 17.9.2 (Medcalc software).

## Results

### Baseline clinical characteristics

**Fig 1:**
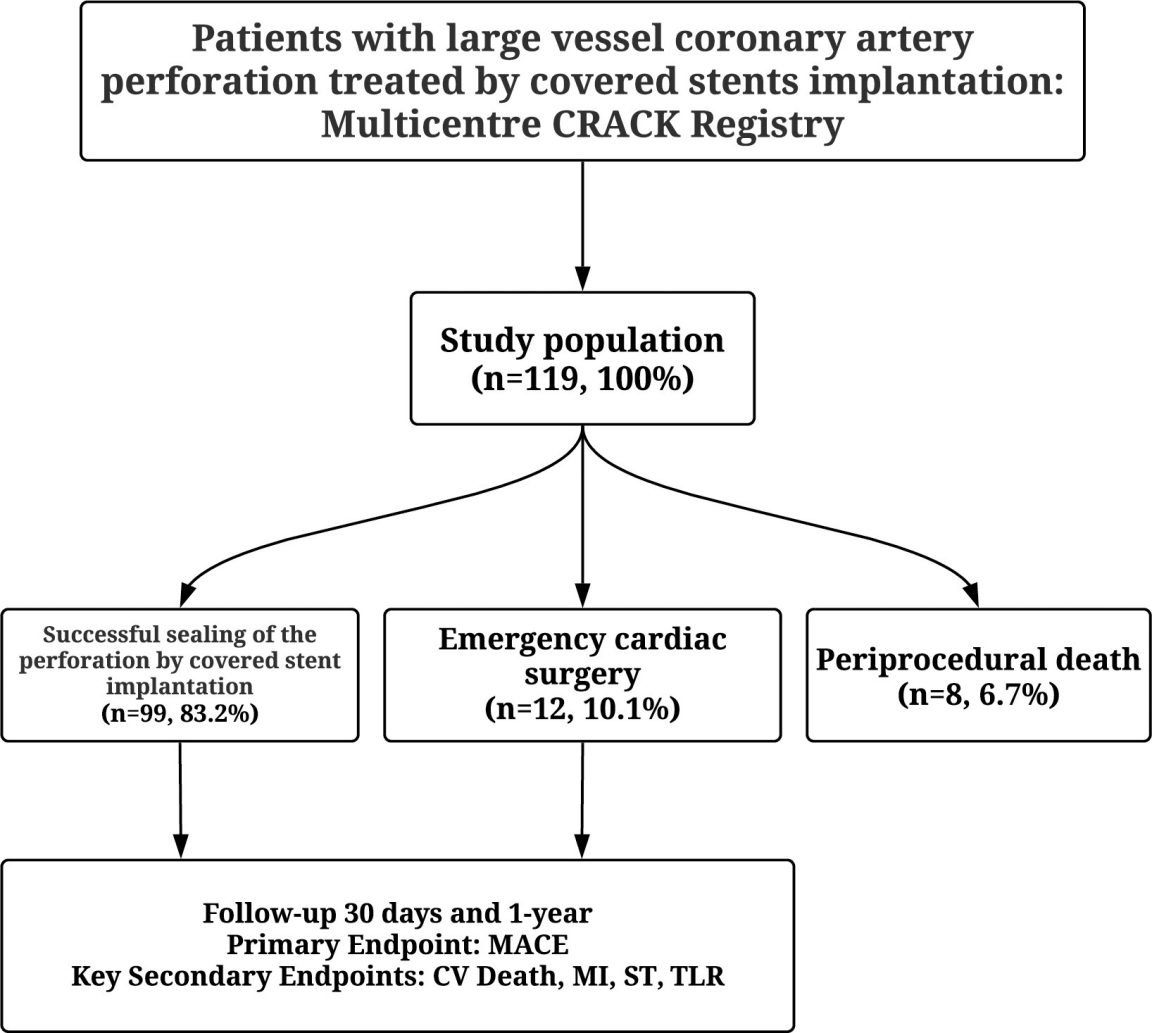
Study flow chart. PCI—percutaneous coronary intervention; CV- cardiovascular; MACE- major adverse cardiac events; MI- myocardial infarction; ST- sent thrombosis; TLR- target lesion revascularization.

The multicentre CRACK Registry enrolled 119 patients (**Fig 1**). The mean age was 68.9 ± 9.7 years, and 66 (55.5%) patients were men. ACS was the presenting diagnosis in 73 (61.3%) of cases [21 (17.6%) unstable angina, 26 (21,8%) NSTEMI, 26 (21.8%) STEMI] and chronic coronary syndromes in 46 (38.7%). Forty three (36,1%) patients had a history of MI, 45 (37.8%) had a previous PCI, and 15 (12.6%) had a prior coronary artery bypass grafting. The baseline clinical characteristics are presented in **Table 1.**

**Table 1:** The baseline clinical characteristics of the patients.

	n = 119 (100%)
Age, mean, SD	68.9 ± 9.7
Male gender, n (%)	66 (55.5)
Body mass index (kg/m2), mean, SD	26.1±5.2
Presentation diagnosis, n (%)	
CCS	46 (38.7)
UA	21 (17.6)
NSTEMI	26 (21.8)
STEMI	26 (21.8)
Previous MI, n (%)	43 (36.1)
Post PCI, n (%)	45 (37.8)
Post CABG, n (%)	15 (12.6)
Diabetes mellitus, n (%)	34 (28.6)
Hypertension, n (%)	94 (79.0)
Atrial fibrillation, n (%)	24 (20.2)
Chronic kidney disease, n (%)	28 (23.5)
Dyslipidemia, n (%)	80 (67.2)
Peripheral artery disease, n (%)	17 (14.3)
COPD, n (%)	16 (13.4)
Neoplasm, n (%)	8 (6.7)
Smoking, n (%)	39 (32.8)
Cardiac arrest before PCI, n (%)	12 (10.1)
Left ventricular ejection fraction, (%), mean, SD	48.0 ± 12.7

Data are shown as mean ± SD or %. CABG- coronary artery bypass grafting, CCS -chronic coronary syndromes, COPD—chronic obstructive pulmonary disease, SD—standard deviation, UA–unstable angina, NSTEMI—non-ST segment elevation myocardial infarction, STEMI- ST-segment elevation myocardial infarction, PCI—percutaneous coronary intervention, MI- myocardial infarction.

### Angiographic and procedural characteristics

The angiographic and procedural characteristics are summarised in **Table 2**. Procedurally, most often-treated vessel territory was the left anterior descending (LAD) artery 58 (48.7%). According to ACC/AHA, the most common lesion type was C 47 (39.5%). The mean degree of stenosis was 88.3 ± 11.5%, and the mean length of stenosis was 27.5 ± 14.4mm. Complex PCI, such as chronic total occlusion (CTO), tortuosity, bifurcation, or severe calcification, was reported in 73 (61.3%) of cases, respectively. The mechanism of perforation was a balloon dilatation in 17 (14.3%) patients, stent implantation in 99 (83.2%) patients, and rotablation in 13 (10.9%) patients. A total of 52 patients (43.7%) had type 3 Ellis classification and 28 patients (23.5%) had type 2 followed by 39 patients (32.8%) with type 1 perforation. The following CS were used: 55 (46.2%) PK Papyrus (Biotronik, Bülach, Switzerland), 51 (42.9%) Graftmaster (Abbot Vascular, Santa Clara, CA), 2 (1.7%) Direct-Stent (Technologies Inc., St. Paul, MN, USA), 6 (5.0%) BeGraft (Bentley Innomed GmbH, Hechingen, Germany), 5 (4.2%) Aneugraft Dx (ITGI Medical, Or Akiva, Israel) **(Table 2)**. The number of CS per patient was 1.1 ± 0.4, with a mean diameter of 3.2 ± 0.5 and a length of 18.8 ± 4.3mm. Effective perforation sealing was achieved in 83.2%, and angiographic success (TIMI 3) was observed in 84.0%. Intravascular imaging was used in 5% of cases. Coronary dissection or no-reflow phenomenon was observed in 23.5% and 6.7% of cases after PCI, respectively.

**Table 2:** Angiographic and procedural characteristics.

	n = 119 (100%)
Radial access, n (%)	77 (64.7)
**Angiography, n (%)**	
1VD	53 (44.5)
2VD	43 (36.1)
3VD	22 (18.5)
**Perforated vessel, n (%)**	
LM	5 (4.2)
LAD	58 (48.7)
LCx	22 (18.5)
RCA	31 (26.1)
SVG	9 (7.6)
**Lesion classification according to ACC/AHA, n (%)**	
Type A	4(3.4)
Type B	37(31.1)
Type B/C	31(26.1)
Type C	47 (39.5)
**Ellis classification, n (%)**	
Type 1	39 (32.8)
Type 2	28 (23.5)
Type 3	52 (43.7)
**Type of stenosis, n (%)**	
De-novo	109 (91.6)
In stent restenosis	10 (8.4)
Chronic total occlusion	7 (5.9)
Bifurcation	17 (14.3)
Severe calcification	36 (30.3)
Tortuosity	13(10.9)
Degree of stenosis, (%), mean, SD	88.3 ± 11.5
Length of stenosis, (mm), mean, SD	27.5 ± 14.4
Lesion predilation, (%)	100 (84.0)
Predilation balloon maximal pressure (atm.), mean, SD	15.1 ± 4.6
Stent deployment maximal pressure (atm.), mean, SD	14.8 ± 2.9
Maximal stent diameter (mm), mean, SD	3.4 ± 0.8
Total stent length (mm), mean, SD	26.5 ± 8.6
Stent postdilation (%)	41 (34.5)
**Direct cause of perforation, n (%)**	
Balloon dilatation	17 (14.3)
Drug eluting stent implantation	83 (69.7)
Bare metal stent implantation	11 (9.2)
Bioresorbable vascular scaffolds implantation	5 (4.2)
Rotablation	13 (10.9)
**Treatment of rupture before cover stent implantation, n (%)**	
Prolonged balloon dilatation	40 (33.6)
Transcatheter fat embolization	2 (1.7)
Non covered stent implantation	10 (8.4)
**Type of covered stent, n (%)**	
Graftmaster	51 (42.9)
Direct-Stent	2 (1.7)
BeGraft	6 (5.0)
PK Papyrus	55 (46.2)
Aneugraft Dx	5 (4.2)
**Covered stent data, mean, SD**	
Length, (mm)	18.8 ± 4.3
Diameter, (mm)	3.2 ± 0.5
Pressure, (atm)	15.2 ± 4.1
Inflation time, (s)	21.0 ± 24.8
Number of covered stent per patient, mean, SD	1.1 ± 0.4
Number of non covered stents per patients, mean, SD	1.2 ± 0.9
Vascular complication, n (%)	
Dissection	28 (23.5)
No-reflow	8 (6.7)
TIMI-3 post PCI, n (%)	100 (84.0)
IVUS, n (%)	6 (5.0)
Glycoprotein IIb/IIIa inhibitors administration, n (%)	10 (8.4)
Protamine sulphate administration, n (%)	20 (16.8)

VD- vessel disease, LM- left main, LAD- left anterior descending artery, LCx- left circumflex artery, RCA- right coronary artery, SVG–saphenous vain graft, SD—standard deviation, PCI—percutaneous coronary intervention, TIMI—Thrombolysis In Myocardial Infarction, IVUS- intravascular ultrasound.

### Periprocedural data

The periprocedural data are summarised in **Table 3**. A total of 46 (38.7%) patients developed cardiac tamponade either during or post-procedure. However, the pericardiocentesis was performed in 45 (37.8%) patients. Cardiogenic shock was observed in 30 (25.2%) patients. Notably, emergency cardiac surgery was performed in 14 (11.8%) patients. Six patients, during the procedure, required the use of mechanical circulatory support.

**Table 3:** Peri-procedural data.

	n = 119 (100%)
Cardiogenic shock, n (%)	30 (25.2)
Pericardial tamponade, n (%)	46 (38.7)
Periprocedural cardiac arrest, n (%)	19 (16.0)
**Adjunct therapy, n (%)**	
Urgency blood transfusion	22 (18.5)
Pericardiocentesis	45 (37.8)
Mechanical circulatory support	6 (5.0)
Dual antiplatelet therapy duration (months), mean, SD	10.6 ± 3.3

### Clinical endpoints

**Fig 2:**
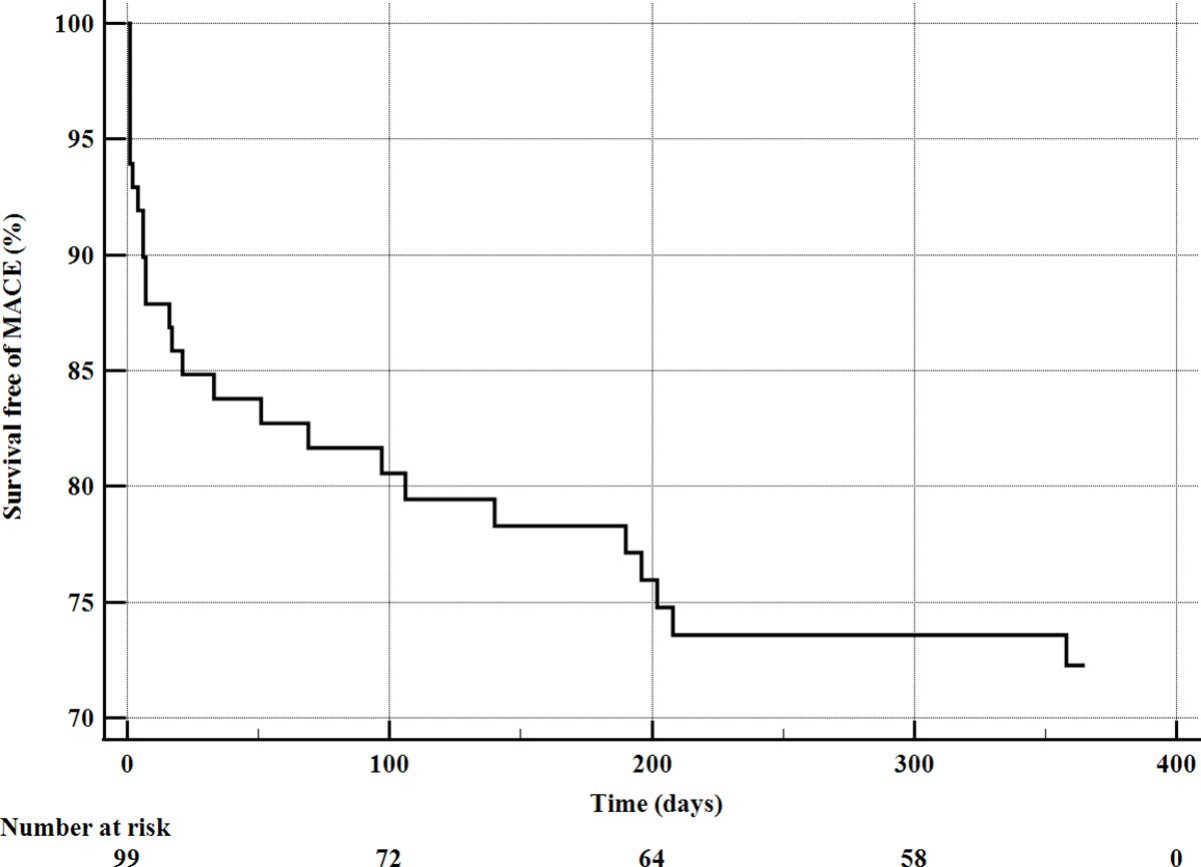
Kaplan–Meier curves for MACE in patients with CAP treated by CS implantation. MACE- major adverse cardiac events.

The clinical study endpoints are summarised in **Table 4** and **Fig 1**. Periprocedural death was observed in eight patients (6.7%), of which two patients required emergency cardiac surgery. Those patients were excluded from the one-year analysis. Successful sealing of a CAP with a CS implantation was achieved in 99 (83.2%) patients. Twelve (10.1%) patients had persistent bleeding despite CS implantation and had emergency cardiac surgery. Cumulative 30-day MACEs were observed in 15 (15.1%) patients. Of those 6 (6.1%) patients experienced cardiac death, 8 (8.1%) TLR, and 6 (6.1%) MIs. Furthermore, four (4.0%) patients had acute stent thrombosis (ST), and one patient (1.0%) had subacute ST. During 12-months follow-up, the cumulative MACEs were observed in 26 (26.2%) patients, of those 7 (7.1%) patients experienced cardiac death, 13 (13.1%) TLR, 11 (11.0%) MIs. One patient (1.0%) had late ST. **Fig 2** shows the Kaplan Meier curve for MACE in patients who received CS for treating CAPs. The subanalysis of type of CS used and 1-year follow-up showed a lower incidence of MI in PK Papyrus CS compared to the Graftmaster CS [2 (4.3%) vs. 7 (17.1%), p = 0.014] **(S1 Table)**. In the first 30-day follow-up, only one TLR was observed in patients who underwent emergency cardiac surgery. During 12-month follow-up, the cumulative MACEs were observed in 2 (14.2%) patients, of those 1 (7.1%) patient experienced TLR, and one (7.1%) had MI.

**Table 4:** 30-days and 1-year follow up after successful sealing by CS implantation (n = 99).

	30 days follow up	1 year follow up
Acute ST, n (%)	4 (4.0)	4 (4.0)
Subacute ST, n (%)	1 (1.0)	1 (1.0)
Late ST, n (%)	-	1 (1.0)
TVR, (%)	8 (8.1)	15 (15.2)
TLR, (%)	8 (8.1)	13 (13.1)
Cardiac death, (%)	6 (6.1)	7 (7.1)
MI, (%)	6 (6.1)	11 (11.0)
MACE, (%)	15 (15.1)	26 (26.2)

ST- sent thrombosis; TVR- target vessel revascularization; TLR- target lesion revascularization, CV- cardiovascular, MI- myocardial infarction, MACE: major adverse cardiac events.

## Discussion

To our knowledge, the current report is one of the largest analyses presenting in-hospital and one-year clinical outcomes following CAPs during PCI treated by CS implantation. The main findings of the present study can be summarized as follows: 1) CS implantation provides prompt and effective treatment of CAP; 2) 83% of perforations were successfully sealed; 3) the use of CS post-CAPs heralds a relatively elevated risk of stent thrombosis and MACE at one year of follow-up.

The CAP is a challenging PCI complication associated with significant morbidity and mortality. A number of previous reports addressed the prevalence and the risk profile predisposing to CAPs [2, 7]. The combination of multiple factors contributes to the increased risk of CAP and the subsequent adverse events such as pericardial effusion or cardiac tamponade: female sex, older age, calcified coronary arteries, complex coronary lesions, previous coronary artery bypass grafting, or rotational atherectomy procedures [7, 16–18]. Studies assessing CAP presented significantly higher morbidity and mortality compared to an uncomplicated PCI, with a potential of an improvement of outcomes post-CS implantation [5, 8, 19]. The implantation of CS is a standard of care in patients with persistent CAP bleeding. However, while CSs were found to terminate CAP bleeding, at the same time (probably due to its delayed endothelialisation), it increases the thrombotic risk, and in particular, ST. Additionally, in the CAP PCI, the ST risk is generally higher than during non-CAP PCI, due to the presence of complex lesions [20]. The optimal management of CAP patients is maintaining a balance between bleeding risk and the risk of CS thrombosis. Still, the stent implantation in CAP can be a life-saving procedure, as compared with emergency cardiac surgery. In the pre-CS era, where cardiac surgery was required in 50–65% of the CAP cases, and 20% of them resulted in death [14]. Large CAPs treated by CS implantation pose an exceptionally high risk of ST, target lesion failure, and MACE. Al-Lamee et al. [21] reported an incidence of definite stent thrombosis of 8.6% (4/46 patients at three years of follow-up) and MACE of 41.3% (19/46 patients at three years of follow-up) in grade III CAP patients treated with CS [21]. In another study Kawamoto et al. [22] evaluated the 57 patients with CAP treated with CSs implantation. The MACE rates were 28% at 30 days, 22% at 1 year, and 38% at 3 years. The rates of target lesion revascularization were 8% and 12% at 1 and 3 years, respectively, which was in accordance with other contemporary big data registers. Harnek et al. [5], in the subgroup analysis of the CSs used in the SCAAR Registry, reported 166 patients post CAP treated by CS. During a one-year follow-up, they found a high number of adverse events with 3.6% of ST and 34.3% of MACE, respectively. In another study, Hachinohe et al. [19] assessed CSs performance in 53 CAPs lesions, with 81% of Graftmaster (Abbot Vascular, Santa Clara, CA) stents used. During a 1-year follow-up, the authors noted 19.6% TLRs, followed by 41.6% at 10-years and 11.9% STs at one year followed by 23.9% at 10-years. Notably, the adverse events frequently occurred in the first year and gradually increased through follow-up. Timing of these events were in line with previous studies in our registry, with 6.1% ST and 26.2% with MACE during a one-year follow-up. Indeed, Jurado-Román et al. [23] presented the homogenous group of 52 patients treated with PK Papyrus CS (Biotronik, Bülach, Switzerland) in CAPs in long term follow up (mean 22±16 months) and, in contrast to other studies, revealed no ST and low incidence of MACE 4 (7.7%). PK Papyrus CS represents a new generation of CS, with a covered single stent based on the Orsiro / PRO-Kinetic Energy platform (Biotronik, Bülach, Switzerland). It has a polyurethane membrane with a thickness of 90 μm. This construction, physiochemical and mechanical properties improves flexibility and may reduce the incidence of ST. In our study we also observed the reduction of the incidence of MI in PK Papyrus CS compered to Graftmaster CS. Previous published series on coronary perforation with covered stent implantation are demonstrated in **Table 5**. In case of CAP, it is difficult to establish whether the increased thrombogenicity potential is only due to the CS or the overlap of drug-eluting stents and CS; the vascular damage was usually associated with manipulation around the stenosis, and the majority of causes of CAP was stent implantation. Notably, it has been proven that suboptimal PCI results in stent underexpansion, struts malapposition, edge dissections, and residual lesions of the treated territory all of which have been well-known risk factors of ST [24, 25]. Probably intravascular imaging with intravascular ultrasound or optical coherence tomography should be performed in those cases to avoid suboptimal PCI results. The need to have life saving devices like covered stents available in the cath lab at all times in order to bail out patients who suffer fatal complications like perforation during PCI and avoid emergency surgery. This is also represented by our data—every third patient required pericardiocentesis, every fifth, blood transfusion, and every tenth emergency cardiac surgery. Emergency cardiac surgery is burdened with high risk of cardiovascular complications. The costs of surgery and hospitalization at the cardiac intensive care unit, together with the risk of periprocedural complications, significantly increases the risk of adverse clinical outcomes, further rehabilitation, and decreased quality of life. That is why it is worthy to invest in technologies and in the training of operators because a high percentage of CAP was successfully managed with the endovascular method. Our study also presents 30-day and one-year follow up data. Clinical events occurred predominantly in the acute and subacute period, up to 30 days after the index procedure. Favourable long-term outcomes of the CS, as demonstrated in our study, suggest that CS implantation in the course of CAP is a safe solution for the treatment of this life-threatening complication. Taking into account the effectiveness of the procedure performed, the number of patients that required cardiac surgery, and the long-term follow-up, the CS implantation in the course of CAP is a safe solution for the treatment of a life-threatening complication.

**Table 5:** Previous published series on coronary perforation with covered stent implantation.

Study	Enrolment years	No of patients with perforation	Percentage of covered stent implantation	Covered Stent type	Success rate of hemostasis	Follow-up	Outcomes						
							Data available for the covered stent population	MACE	Stent thrombosis	TVR	TLR	Death	MI
Kawamoto et al. 2015 [22]	2004–2015	285	20%	ePTFE	88%	30-days	YES	28%	0%	2%	2%	7%; 2%‡	18%
						6-months		16%	2%	11%	8%	6%; 4%‡	2%
						1-year		22%	2%	17%	8%	6%; 4%‡	2%
						2-years		32%	5%	22%	8%	11%; 9%‡	5%
						3 years		38%	5%	26%	12%	17%; 15%‡	5%
Chen et al. 2015 [26]	2008–2013	9	100%	Processed equine pericardium	100%	Up to 32 months	YES	NA	11%	44% (1-ST; 2-ISR; 1- occluded)		0%	22%
Lee et al. 2016 [27]	2004–2016	48	100%	ePTFE	100%	30 days	YES	NA	0%	NA	0%	16.7%; 13%‡	0%
						1 year		NA	16%	NA	22%	26%; 22%‡	6%
						3 years		NA	22%	NA	52%	38%; 29%‡	9%
Lemmert et al. 2017 [7]	2005–2016	150	24%	NA	NA	In hospital	YES	NA	NA	NA	NA	14%	NA
						30 days		NA	NA	NA	NA	25%	NA
						1 year		NA	NA	NA	NA	37%	NA
Guttmann et al. 2017 [28]	2005–2016	149	21%	NA	NA	In hospital	YES	NA	NA	NA	NA	16%	NA
						1.4 year (median)			29.6%			NA	
Mirza et al. 2018 [29]	2009–2016	24	62.5%	ePTFE	NA	2 years	YES	NA	6.7%	NA	NA	0%	0%
Kufner et al. 2018 [30]	2013–2017	61	100%	ePTFE	96.7%	In-hospital	YES	NA	0%	NA	0%	8.2%; 8.2%‡	31.1%
						<1 year		NA	0%	NA	18.0%	11.5%; 8.2%‡	31.1%
Kandzari et al. 2019 [31]	2013–2017	80	100%	Electrospun polyurethane	91.3%	In-hospital	YES	NA	1.2%	NA	NA	10%	0%
Hachinohe et al. 2019 [19]	1997–2007	53	100%	ePTFE, Processed equine pericardium	NA	1 year	YES	NA	11.9%	18.8% ^	19.6%	4.2‡	13.5%*
						5 years			23.9%	38.4%^	37.7%	10.2%‡	20.6%*
						10 years			23.9%	38.4%^	41.6%	14.4%‡	20.6%*
Rossel et al. 2019 [32]	2007–2017	55	43.6%	ePTFE, Processed equine pericardium	95.8%	In-hospital	YES	NA	NA	NA	NA	8.3%	NA
						1 year		23.8%	0%	NA	NA	9.5%	NA
						5 years		58.8%	4.2%	NA	NA	26.7%	NA
Parikh et al. 2019 [17]	2003–2017	67	35.8%	ePTFE	NA	30 days	YES	NA	0%	NA	0%	8%	NA
						1 year		NA	NA	NA	4.2%	16.7%	NA
						4 years		NA	NA	NA	12.5%	50%	NA
Itoh et al. 2020 [33]	2003–2014	82	18.3%	ePTFE	73.3%	In-hospital	YES	NA	NA	NA	NA	26.7%	13%
Cerrato et al. 2020 [34]	1998–2018	311	48.2%	NA	84%	In-hospital	NO	38.2%	2.2%	NA	2.9%	6.1%	28.3%
Harnek et al. 2020 SCAAR Registry [5]	2005–2017	1008	16.5%	ePTFE, Processed equine pericardium, Electrospun polyurethane	NA	1 year	YES	34.3%	3.6%	NA	10.2%	19.3%	7.8%
Hernández-Enríquez et al. 2020 [35]	2012–2017	61	100%	ePTFE, Electrospun polyurethane	75%	In-hospital	YES	31%	2%	NA	0%	18%	7%
						1 year		57%	4%	10%	7%	36%; 33%‡	9%
Jurado-Roman et al.2020 [23]	2014–2019	52	100%	Electrospun polyurethane	94.2%	22 ± 16 months	YES	7.7%	0%	NA	3.8%	3.8% ‡	3.8%*
Wańha et al.2021 CRACK Registry	2009–2019	119	100%	ePTFE, Processed equine pericardium, Electrospun polyurethane	83%	30 days	YES*	15.1%	5%	8.1%	8.1%	6.1%‡	6.1%
						1 year		26.2%	6.1%	15.2%	13.1%	7.1%‡	11.0%

MACE- major adverse cardiovascular events; ST- stent thrombosis; TVR- target vessel revascularization; TLR- target lesion revascularization; MI- myocardial infarction

*Periprocedural death and patients required emergency cardiac surgery were excluded from the one-year analysis

‡cardiac death

^target vessel occlusion

*Target vessel MI.

### Study limitations

There are several limitations to this study. First, we had no data on intravascular imaging data, and thus the mechanism of recorded ST is unknown. Second, there was a lack of quantitative findings such as reference vessel diameter or minimal lumen diameter.

## Conclusions

Implantation of CS is a successful treatment of CAPs. This real-world data on CAPs treated with covered stents illustrated that the procedural and 1-year outcomes of CAP treated by CS implantation warrant diligent follow-up of the patients as they are at increased risk of MACE. Events of ST warrant further studies and use of intravascular imaging to optimize the CS implantation may be indicated.

### Learning objectives

Coronary artery perforation (CAP) can be a life-threatening complication of percutaneous coronary intervention, which requires a rapid treatment. The covered stents (CS) emerge as a promising solution that can successfully seal the perforation in 83% of cases. All post-CAP patients treated by CSs implantation should be closely followed due to high risk of stent thrombosis or other adverse cardiac events.

## Supporting information

S1 Table Subanalysis of type of covered stent uses 1-year follow up (n = 99, 100%). (DOCX)
