# Supplementary material for: Procedural and 1-year outcomes following large vessel coronary artery perforation treated by covered stents implantation: Multicentre CRACK registry
Source: PLoS One. 2021 May 12;16(5):e0249698. doi: 10.1371/journal.pone.0249698 (PMC8115813; doi:10.1371/journal.pone.0249698)
Supplement: S1 Table — (DOCX) [file pone.0249698.s001.docx]

**S1 Table. Subanalysis of type of covered stent uses 1-year follow up (n=99, 100%).**

| n (%) | Aneugraft 4 (4.0) | BeGraft 6 (6.1) | Direct Stent 1 (1.0) | Graftmaster 41 (41.4) | Pk Papyrus 47 (47.5) | p |
| --- | --- | --- | --- | --- | --- | --- |
| Acute ST, n (%) | - | - | - | 3 (7.3) | 1 (2.1) | 0.728 |
| Subacute ST, n (%) | - | - | - | 1 (2.4) | - | 0.839 |
| Late ST, n (%) | - | - | - | 1 (2.4) | - | 0.839 |
| TVR, n (%) | - | - | - | 9 (22.0) | 6 (12.8) | 0.455 |
| TLR, n (%) | - | - | - | 8 (19.5) | 5 (10.6) | 0.496 |
| CV death, n (%) | - | - | - | 1 (2.4) | 6 (12.8) | 0.343 |
| MI, n (%) | - | 1 (16.7) | 1 (100) | 7 (17.1) | 2 (4.3) | 0.014 |
| MACE, n (%) | - | 1 (16.7) | 1 (100) | 12 (29.2) | 12 (25.5) | 0.317 |
|  | | | | | | |

ST- sent thrombosis; TVR- target vessel revascularization; TLR- target lesion revascularization, CV- cardiovascular, MI- myocardial infarction, MACE: major adverse cardiac events.
